# Cross-Layer Protocol Design and Performance Evaluation of LoRa Ad Hoc Networks for Heterogeneous Traffic

**DOI:** 10.3390/s26154718

**Published:** 2026-07-24

**Authors:** Shengli Pang, Yuanyuan Ma, Xianjin Cheng, Fan Yang, Zimiao Zou, Ruoyu Pan, Honggang Wang

**Affiliations:** College of Communication and Information Engineering, Xi’an University of Posts and Telecommunications, Xi’an 710121, China; pangshengli@xupt.edu.cn (S.P.); chengxianjin@stu.xupt.edu.cn (X.C.); yangfan@stu.xupt.edu.cn (F.Y.); zouzimiao@stu.xupt.edu.cn (Z.Z.); panruoyu@xupt.edu.cn (R.P.); wanghonggang@xupt.edu.cn (H.W.)

**Keywords:** LoRa ad hoc networks, relay deployment, MAC, cross-layer routing, heterogeneous services

## Abstract

To address the severe coverage blind spots and concurrent collision bottlenecks faced by LoRa networks in dense deployments and complex three-dimensional (3D) occlusion environments, this paper proposes a distributed cross-layer protocol framework for LoRa ad hoc networks supporting heterogeneous traffic. To break through the limitations of a single star architecture, this framework constructs a 3D penetration loss model at the physical layer and designs a distributed relay deployment algorithm based on hybrid simulated annealing, achieving blind-spot-free connectivity in complex spaces. At the MAC layer, a non-preemptive priority access mechanism based on symbol energy detection is introduced. Through differentiated backoff windows with time-domain isolation, it precisely guarantees the quality of service (QoS) requirements of heterogeneous traffic and significantly suppresses concurrent collisions. At the network layer, the CAM-AODV routing algorithm is proposed, which integrates hop count, link quality, MAC queue congestion, and nodal residual energy to achieve dynamic traffic diversion and network-wide energy balancing under bursty high loads. Simulation results demonstrate that this cross-layer framework effectively breaks the traditional network capacity bottlenecks. In a large-scale, high-density scenario with 300 nodes, CAM-AODV reduces the average end-to-end delay by 19.46% compared to the traditional AODV. Under high-concurrent loads, the packet delivery ratio (PDR) of the proposed framework improves by 16.32% over the traditional protocol, while the system delay is reduced by 13.66%. Furthermore, under the two aforementioned evaluation scenarios, the Energy Balancing Index (EBI) is significantly improved by 11.13% and 10.57%, respectively, compared to the traditional protocol. This study provides an efficient joint optimization scheme for building high-capacity, wide-coverage, and long-lifespan complex Internet of Things (IoT) networks.

## 1. Introduction

In recent years, driven by the accelerated deployment of Low-Power Wide-Area Networks (LPWANs) in domains such as smart cities and industrial monitoring, LoRa, as a prominent representative, has attracted widespread attention from academia owing to its long-range and low-power characteristics. However, Adelantado et al. deeply analyzed the theoretical limits of standard LoRaWAN, explicitly pointing out that under dense node deployment scenarios, the concurrent access of massive end nodes frequently triggers severe co-channel collisions, whereby the single-hop star architecture becomes a major bottleneck restricting network scalability [1]. Furthermore, Pham et al. conducted a comprehensive evaluation of Channel Activity Detection (CAD) and the capture effect in dense LoRa networks, further confirming that the packet delivery ratio (PDR) of the pure star architecture experiences a precipitous decline when handling high-concurrent traffic [2].

Regarding network architecture and relay deployment, Cotrim et al. systematically demonstrated the feasibility of LoRa multi-hop architectures, proposing that introducing a mesh ad hoc network is an effective approach to extending network coverage [3]. Building upon this, García et al. verified the seamless coexistence of a “star-mesh” hierarchical hybrid topology, demonstrating that this architecture can significantly expand the communication range while remaining compatible with existing protocols [4]. To address the issue of coverage blind zones in multi-hop networks, Haif et al. proposed a distributed dynamic relay deployment framework, which maximizes the link signal-to-noise ratio (SNR) by optimizing the geometric locations of nodes [5]. Additionally, Lee et al. explored the design and evaluation of utilizing an LoRa wireless mesh network system for large-scale IoT sensor monitoring, further corroborating the potential of relay deployment in extending coverage [6]. However, most existing relay deployment schemes are confined to ideal two-dimensional planes, and rarely incorporate complex three-dimensional (3D) spatial penetration fading into their optimization considerations.

In terms of channel access and anti-collision mechanisms, standard LoRaWAN defaults to a pure ALOHA random access strategy without carrier sensing, which leads to drastically deteriorating channel collisions when the network load surges. To address this, Leonardi et al. conducted a detailed comparison between ALOHA and the carrier-sensing-based Listen-Before-Talk (LBT) mechanism. Their experiments verified that LBT can substantially reduce the packet collision rate in high-concurrency scenarios [7]. Subsequently, Mehling et al. investigated the parameter configuration of LBT backoff windows in multi-gateway environments [8], while Cao et al. provided an in-depth analysis of the hidden end nodes probability distribution across annular regions defined by different spreading factors (SFs) in single-gateway scenarios [9]. These studies lay a crucial foundation for designing lightweight anti-collision mechanisms. Furthermore, to accommodate emergency applications such as fire early warnings, Jain et al. proposed the Burst-MAC protocol specifically tailored for bursty traffic, which utilizes semi-distributed scheduling to handle instantaneous high-concurrent data [10]. However, most existing LBT anti-collision mechanisms are designed for single-traffic scenarios and lack quality of service (QoS) isolation mechanisms for heterogeneous traffic. Consequently, they remain highly susceptible to queue overflow under extremely high-concurrency conditions.

Focusing on the design of multi-hop ad hoc routing protocols, Lundell et al. pioneered the introduction of the Ad hoc On-Demand Distance Vector (AODV) routing protocol into LoRa mesh networks, validating its fundamental feasibility in low-data-rate networks [11]. To further mitigate the control packet storms frequently triggered by traditional AODV during the route discovery phase, Galzarano et al. proposed a Gossip-based AODV protocol, effectively reducing flooding overhead in wireless sensor networks by incorporating a probabilistic forwarding model [12]. However, whether utilizing the traditional AODV or its lightweight variants, the reliance on a single “minimum hop count” metric remains highly prone to causing energy depletion at core nodes or localized congestion. Consequently, routing decisions are progressively evolving towards cross-layer multi-metric approaches. During the route discovery phase, Macaraeg et al. and Chen et al. incorporated the Received Signal Strength Indicator (RSSI) and node congestion levels, respectively. These integrations effectively circumvent poor physical links and enhance the reliability of emergency communications [13,14]. Tailored for large-scale constrained networks, Udugampola et al. and Misbahuddin et al. proposed novel routing strategies based on node location, residual energy, and low-latency awareness, respectively. These strategies effectively balance global energy consumption and prolong network lifetime [15,16]. Regarding heterogeneous traffic processing, Zhou investigated routing protocols for large-scale sensor networks supporting mixed traffic, emphasizing the importance of differentiated routing for data streams with diverse QoS requirements [17]. Meanwhile, Chou et al. proposed a cross-layer AODV protocol integrated with MAC-layer scheduling, verifying that the underlying queue state exerts a significant positive feedback effect on upper-layer routing decisions [18]. Chong et al. explored an AODV-inspired routing mechanism featuring next-hop caching and path selection capabilities [19]. For emergency alert systems, Thaenthong et al. proposed a Reverse Ad-hoc On-Demand Distance Vector (R-AODV) routing algorithm based on LoRa mesh networks [20]. With respect to energy efficiency and global balancing, Medeiros et al. reviewed energy-efficient routing protocols in smart cities [21], while Scarvaglieri et al. presented a distributed Multi-Armed Bandit (MAB)-based algorithm designed for energy-fair and reliable routing in multi-hop LoRa networks [22]. In recent years, driven by the growing complexity of IoT services, artificial intelligence (AI) technologies have been increasingly introduced into cross-layer routing design. For example, targeting hybrid IoT systems, Karthi et al. proposed an intelligent cross-layer routing protocol based on multi-agent actor–critic reinforcement learning, demonstrating the immense potential of multi-dimensional state fusion in enhancing network reliability [23]. Furthermore, in practical application deployments, Vel Murugan et al. designed a smart energy metering and secure billing system for remote rural areas based on the LoRa network architecture. They proposed a multi-hop routing and data aggregation strategy tailored for wide-area sparse nodes, further validating the application value of ad hoc routing protocols in practical constrained environments [24]. Similarly, Sim et al. explored the application of LoRa mesh ad hoc algorithms in large-scale remote aquaculture monitoring, further corroborating the high energy efficiency and reliability of AODV multi-hop routing in wide-area, long-range data collection [25]. Although the aforementioned studies have made preliminary progress in multi-dimensional routing metrics or isolated MAC-layer queue feedback, existing protocols still struggle to achieve deep cross-layer integration of multi-dimensional indicators—such as the underlying queue congestion state, physical-layer link quality, and network-layer topology—in dense deployment scenarios. Consequently, when confronted with high-concurrent heavy traffic, they are highly susceptible to queue congestion and packet overflow drops at local relay nodes.

To intuitively illustrate the positioning of this study within the existing literature, Table 1 provides a comparative summary of several representative works and the cross-layer joint optimization framework proposed in this paper.

In summary, while existing research on multi-hop LoRa routing and MAC protocols has yielded abundant results, it primarily focuses on network architecture improvements or single-layer protocol optimizations, making it difficult to thoroughly resolve the complex communication bottlenecks caused by dense deployments and high concurrency. To break through the limitations of traditional designs, this paper constructs a distributed LoRa ad hoc network joint optimization framework encompassing topology deployment and cross-layer collaboration. Compared with existing mature studies, the core innovations and contributions of this paper are mainly reflected in the following three aspects:

1. Proposing a relay optimization deployment algorithm for complex 3D fading. Existing multi-hop coverage schemes are mostly confined to ideal 2D planes, ignoring the severe blockages caused by urban building clusters. This paper innovatively constructs a penetration loss model based on 3D Axis-Aligned Bounding Boxes (AABBs) and employs a hybrid simulated annealing algorithm for relay optimization. This scheme not only eliminates the non-line-of-sight (NLOS) coverage blind spots in 3D physical space but also establishes highly connected backbone backhaul links for long-distance data forwarding.
2. Designing a time-domain isolated MAC access mechanism for heterogeneous traffic. Existing anti-collision mechanisms mostly target homogeneous traffic, which can easily trigger mutual preemption and queue overflows among traffic with different QoS requirements under high concurrency. This paper innovatively designs a non-preemptive priority queuing model. By introducing a differentiated backoff algorithm based on the synergy of symbol energy detection and contention windows, it achieves strict access probability isolation for heterogeneous traffic in the time domain, effectively guaranteeing the low-latency transmission of emergency data and significantly boosting overall network capacity.
3. Constructing a deeply coupled CAM-AODV cross-layer routing protocol. Current AODV improvement schemes generally rely on fixed thresholds or single-link metrics, lacking adaptability to dynamic loads. The proposed CAM-AODV algorithm achieves deep coupling across the physical, MAC, and network layers, dynamically integrating link RSSI, real-time MAC queue congestion, and nodal residual energy into its routing decisions. Through an original non-linear energy and congestion penalty mechanism, the algorithm proactively guides data flows to circumvent local hotspots and near-death nodes in high-concurrency scenarios, flawlessly realizing network-wide dynamic traffic diversion and energy balancing.

The remainder of this paper is organized as follows: Section 2 constructs the system model for the distributed LoRa network, elaborating on the discrete-state energy consumption model, multi-dimensional composite fading characteristics, and the capture effect of concurrent collisions. Section 3 details the proposed cross-layer ad hoc protocol framework, including the hybrid simulated annealing-based relay deployment algorithm, the non-preemptive priority MAC access mechanism, and the improved CAM-AODV cross-layer multi-metric routing algorithm. Section 4 comprehensively evaluates and comparatively analyzes the performance of the proposed algorithms from multiple dimensions, such as 3D coverage, network density, and traffic load, via a MATLAB (version R2022b, The MathWorks, Inc., Natick, MA, USA) simulation platform. Section 5 concludes the paper.

## 2. System Model

### 2.1. Network Model

**Figure 1:**
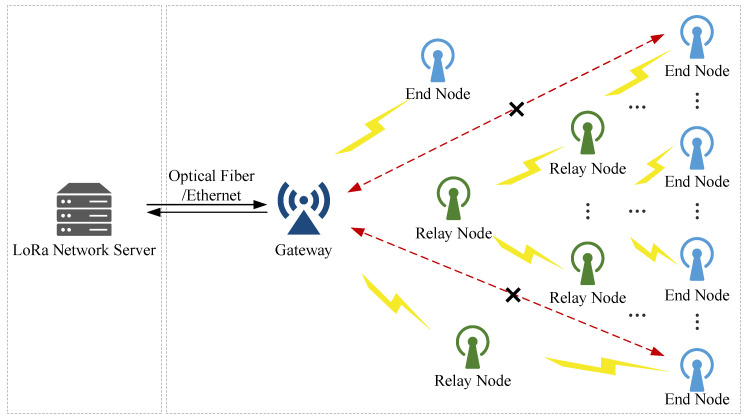
Distributed LoRa network architecture. The yellow lightning symbols denote successful communication links, the red dotted lines with an ‘x’ represent failed communication links.

To address communication dead zones in complex environments, this paper overcomes the limitations of the traditional single-hop star architecture in LoRaWAN by adopting a hierarchical networking architecture that integrates star and mesh topologies. This design enhances network coverage through the hierarchical collaboration of end nodes access, relay forwarding, and gateway aggregation. The overall distributed LoRa network architecture is illustrated in Figure 1. Within this distributed framework, end nodes located inside the effective coverage area of a gateway transmit their data directly to the gateway via a single hop. Conversely, if direct communication is hindered by obstacle occlusion or signal interference, the end nodes utilize a routing algorithm to select the optimal relay node for multi-hop forwarding until the data successfully reaches the gateway. Throughout this process, relay nodes serve as the backbone support, providing potential multi-hop physical links for constrained end nodes. Subsequently, the LoRa network server performs centralized deduplication and parsing on the packets aggregated from multiple paths, ultimately forwarding them to the application server [26]. Given that the deployment of standard gateways relies on a continuous power supply and stable IP backhaul links, the traditional approach of densely deploying multiple gateways to enhance network capacity often encounters significant physical constraints and economic costs in infrastructure-deficient scenarios, such as complex blind zones or emergency communications. Consequently, the proposed network model is designed based on a single-gateway scenario with severely constrained backhaul resources. It primarily investigates how to effectively achieve coverage extension and mitigate concurrent congestion by relying solely on relay nodes through cross-layer multi-hop routing and dynamic traffic diversion mechanisms, thereby ensuring reliable communication in constrained environments. The specific triggering mechanisms for relay nodes, the dynamic classification logic for end nodes, and the optimal spatial deployment algorithm will be elaborately designed and derived in Section 3 of this paper.

### 2.2. Energy Consumption Model

To quantitatively evaluate the network energy consumption and provide constraints for subsequent protocol design, this paper establishes a discrete-state energy consumption model for end nodes.

**Figure 2:**
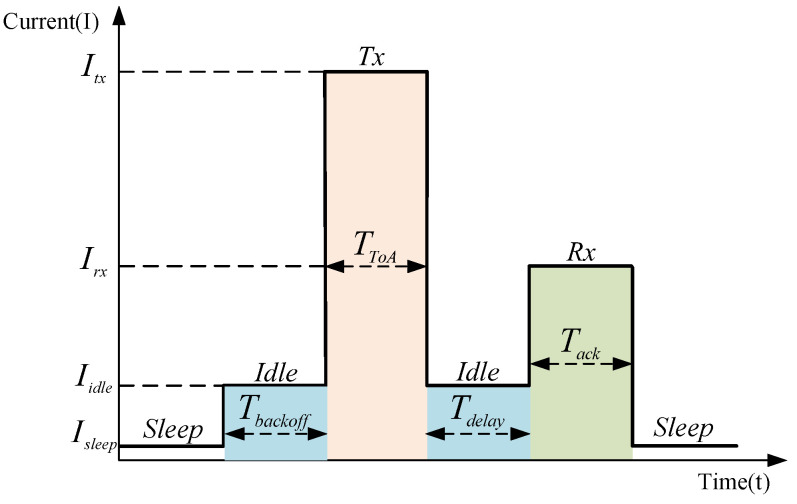
End node discrete state transitions and energy consumption evolution.

As illustrated in Figure 2, the operating cycle of a end node is discretized in the time domain into four physical states: transmission (Tx), reception (Rx), idle, and sleep.

As observed from the figure, during a typical single communication interaction, the node spends the vast majority of its time in the sleep state, operating at near-zero power consumption. Only when triggered by traffic is the node awakened, initially entering the idle state to perform LBT and random backoff. The idle energy consumption generated during this period is highly positively correlated with the degree of network congestion. Upon completing the backoff, the node seizes the channel and transitions into the high-power Tx phase to transmit data. The duration of this phase is primarily dictated by the time-on-air ($T_{\mathrm{ToA}}$), which depends on the target SF and payload size *L*. Following the transmission, the node enters a brief idle waiting period, and subsequently opens its reception window during the Rx phase to await acknowledgments or control signaling. Upon completing the interaction, it ultimately reverts to the sleep state.

Let $V_{\mathrm{sys}}$ denote the operating system voltage of the node, and let $I_{\mathrm{tx}},\;I_{\mathrm{rx}},\;I_{\mathrm{idle}}$, and $I_{\mathrm{sleep}}$ represent the steady-state currents in the transmission, reception, idle, and sleep states, respectively. During a single communication interaction, the core energy consumption incurred by the node in the transmission state can be expressed as follows:

(1) $$E_{\mathrm{tx}}\left(L,\mathrm{SF}\right)=V_{\mathrm{sys}}\cdot I_{\mathrm{tx}}\cdot T_{\mathrm{ToA}}\left(L,\mathrm{SF}\right)$$

where *L* denotes the payload length of the data packet, SF represents the currently applied spreading factor, and $T_{\mathrm{ToA}}\left(L,\mathrm{SF}\right)$ indicates the time-on-air corresponding to this payload and spreading factor.

Similarly, the energy consumption of a node in the reception, idle backoff, and sleep states is linearly determined by the duration and instantaneous power corresponding to each state. Let $E_{\mathrm{init}}$ denote the initial energy reserve of node *i*. Then, at time *t* during network operation, the dynamic residual energy $E_{\mathrm{res}}^{i}\left(t\right)$ of this node can be formulated as an integral model in the time domain:

(2) $$E_{\mathrm{res}}^{i}\left(t\right)=E_{\mathrm{init}}-\int_{0}^{t}P_{i}\left(\tau \right)\;d\tau$$

where $P_{i}\left(\tau \right)$ denotes the instantaneous power of the node corresponding to its state at time τ. This mathematical model explicitly elucidates the dependence of the node’s lifetime on the frequency and duration of physical-layer transmission and reception.

### 2.3. Link Loss and Collision Interference Models

To accommodate the differentiated traffic requirements of massive end nodes in dense deployment scenarios, the system’s communication mechanism supports the orthogonal allocation of multiple channels and spreading factors. By partitioning beacon channels, data uplink channels, and control channels, the system effectively isolates the collision domains of various traffic types at the physical layer. Furthermore, to evaluate the link quality among nodes and network topological connectivity, this paper establishes a channel computation model that comprehensively incorporates large-scale fading, 3D spatial occlusions, and concurrent interference.

#### 2.3.1. Basic Path Loss Model

To address the free-space attenuation characteristics of LoRa signals in complex environments, this paper constructs a large-scale basic path loss model based on the log-distance model. This baseline model is utilized to quantify the signal attenuation between transceiver nodes under line-of-sight (LOS) conditions. The calculation of the basic path loss, denoted as $PL_{\mathrm{base}}$, comprehensively incorporates the Euclidean spatial distance *d* between the transceiver nodes, the carrier center frequency *f*, and the inherent fading characteristics of the environment. Its mathematical expression is defined as follows:

(3) $$PL_{\mathrm{base}}\left(d\right)=20\log_{10}\left(f\right)+10n\log_{10}\left(d/d_{0}\right)+\gamma_{\mathrm{env}}+C$$

where *f* denotes the carrier center frequency allocated to the current channel; *n* represents the basic path loss exponent; $d_{0}$ is the reference distance (typically set to 1 m); $\gamma_{\mathrm{env}}$ indicates the fading margin in the specific deployment environment; and *C* is a constant calibration term.

#### 2.3.2. 3D Spatial Modeling and Penetration Loss Model

**Figure 3:**
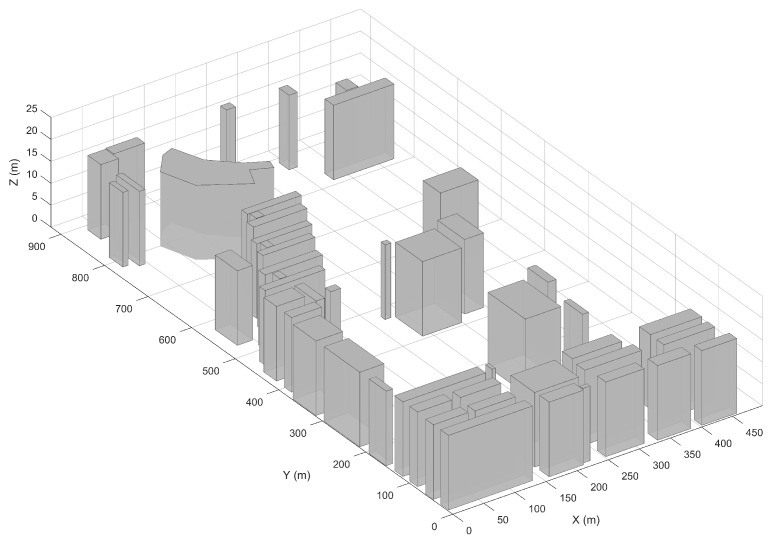
3D spatial model.

To address the NLOS propagation attenuation induced by complex building clusters, this paper conducts a reasonable geometric abstraction of the actual geographical environment. As illustrated in Figure 3, the system employs a hybrid approach combining 3D Axis-Aligned Bounding Boxes (AABBs) and polygonal cylinders to geometrically model the buildings. Let $B=\{B_{1},B_{2},\cdots ,B_{N}\}$ denote the set of buildings, where each building $B_{i}$ is characterized by its set of contour coordinates on the two-dimensional plane and its height attribute $h_{i}$.

Based on the aforementioned 3D spatial model, the system employs a spatial line-segment intersection algorithm to perform connectivity detection on the 3D direct transmission path between the source and destination nodes. If the transmission line segment $\bar{uv}$ spatially intersects with any building $B_{i}$ in set *B*, the communication link is deemed obstructed. In this scenario, an additional penetration loss penalty term, denoted as $PL_{\mathrm{obs}}$, must be superimposed onto the basic path loss to reflect the attenuation of radio frequency (RF) signals caused by building walls. The final RSSI measured at the receiving node *v* is calculated as follows:

(4) $${\mathrm{RSSI}}_{u,v}=P_{\mathrm{tx}}-\left(PL_{\mathrm{base}}\left(d_{u,v}\right)+\sum I_{\mathrm{obs}}\cdot PL_{\mathrm{obs}}\right)$$

where *u* and *v* denote the source and destination nodes, respectively; $P_{\mathrm{tx}}$ represents the transmit power of the node; $d_{u,v}$ indicates the 3D Euclidean spatial distance between the two nodes; $I_{\mathrm{obs}}$ is a Boolean indicator variable, which equals 1 if the transmission path is obstructed and 0 otherwise; and $PL_{\mathrm{obs}}$ denotes the penetration loss penalty term for a single traversal through a building.

To evaluate the quality of NLOS links, the proposed model adopts the layered cumulative framework of the Multi-Wall Model [27]. Considering the physical characteristics of brick-and-concrete buildings, this paper references the statistical distribution of penetration loss for traditional buildings specified in the ITU-R P.2109-2 standard [28]. The typical median value is extracted as a fixed empirical constant for single-wall penetration, thereby facilitating cumulative calculations in complex environments.

**Figure 4:**
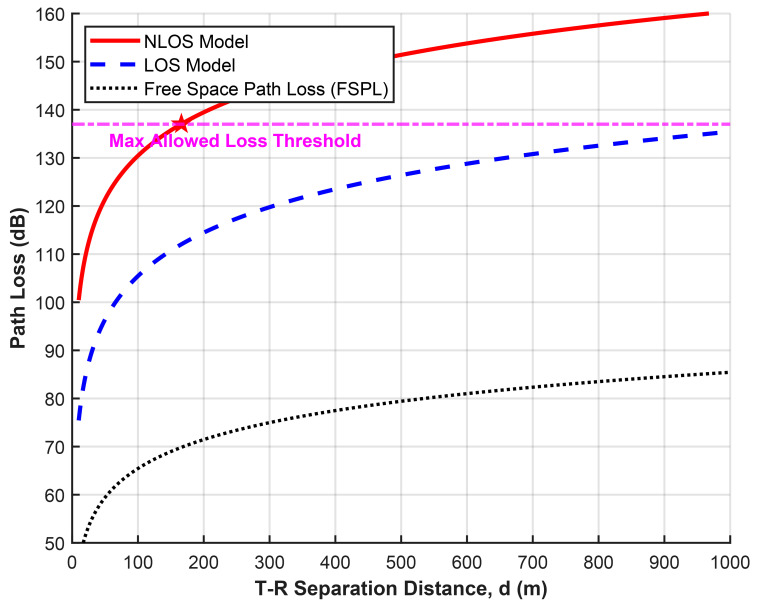
Multi-dimensional composite fading characteristics of signals in complex spatial environments.

Based on the aforementioned basic path loss and 3D penetration fading models, the path loss trends under different spatial LOS conditions are illustrated in Figure 4. In the figure, the NLOS model is superimposed with a building penetration penalty $L_{\mathrm{wall}}$, whereas the LOS model incorporates an environmental fading factor. The pink dashed line represents the maximum allowable path loss threshold of the system. This threshold is calculated as the difference between the node’s transmit power $P_{\mathrm{tx}}$ and the hardware receiver sensitivity $P_{\mathrm{sens}}$, i.e., $\mathrm{MAPL}=P_{\mathrm{tx}}-P_{\mathrm{sens}}$. In the theoretical fading evaluation of this figure, assuming a node transmit power of $P_{\mathrm{tx}}=14\;\mathrm{dBm}$, and under the configuration of SF=7 and a bandwidth BW=125kHz (corresponding to the Semtech SX1276 LoRa transceiver (Semtech Corporation, Camarillo, CA, USA) sensitivity of $P_{\mathrm{sens}}\approx -123\;\mathrm{dBm}$ [29]), this maximum allowable loss threshold is calculated to be 137 dB.

The figure compares three distinct scenarios: ideal Free Space Path Loss (FSPL), LOS basic fading, and NLOS penetration fading. As observed from the figure, under unobstructed LOS conditions, even when the signal transmission distance reaches 1000 m, the path loss remains within the system’s maximum allowable link loss threshold. This demonstrates that the gateway possesses the theoretical capability to achieve wide-area coverage access via a single hop. Conversely, in NLOS scenarios, the link loss escalates significantly due to building penetration loss, causing the signal strength to rapidly attenuate below the spread spectrum receiver sensitivity.

In summary, the complex 3D spatial penetration loss severely constrains the effective coverage range of LoRa links, rendering the conventional single-hop direct star topology inadequate to guarantee network-wide communication reliability and connectivity.

#### 2.3.3. Model of Concurrent Collisions and Capture Effects

**Figure 5:**
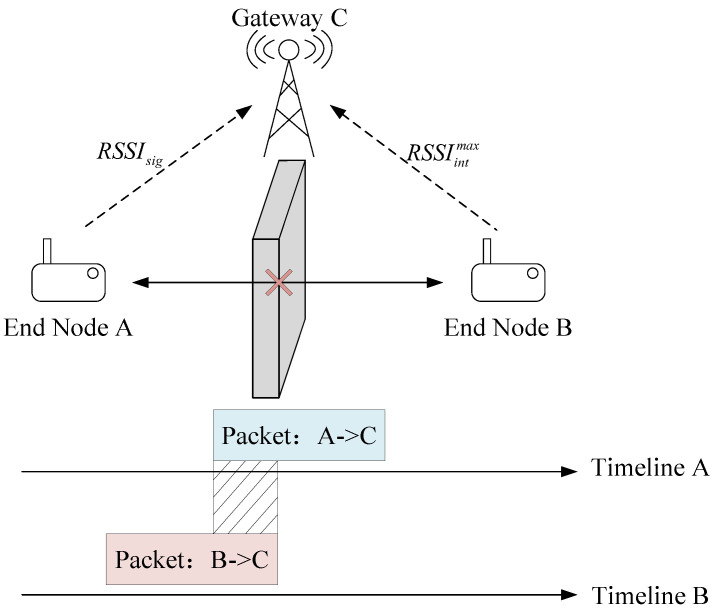
Timing model of hidden end nodes and co-channel interference.

In densely deployed distributed LoRa ad hoc networks, nodes are not only constrained by the constant environmental noise floor, but are also heavily subjected to collision interference caused by hidden end nodes or concurrent co-channel transmissions. As illustrated in Figure 5, when a severe obstruction exists between end node A and end node B, their RF signals are physically blocked from each other, thereby forming a hidden end node topology [9]. If both nodes initiate communication with gateway C at approximately the same time, their data packets will overlap in the time domain at the receiver, resulting in severe co-channel concurrent collisions [2].

To address this, by combining the physical anti-interference characteristics of LoRa’s Chirp Spread Spectrum (CSS) modulation, this paper introduces a capture effect determination model. When multiple signals overlap in the time domain, if the received signal strength of the target signal ${\mathrm{RSSI}}_{\mathrm{sig}}$ is higher than that of the strongest concurrent interfering signal ${\mathrm{RSSI}}_{\mathrm{int}}^{\max}$, and their difference satisfies a specific capture isolation threshold, denoted as $\Delta_{\mathrm{cap}}$, that is,

(5) $${\mathrm{RSSI}}_{\mathrm{sig}}-{\mathrm{RSSI}}_{\mathrm{int}}^{\max}\geq \Delta_{\mathrm{cap}},$$

then the receiver can successfully capture the target signal at the physical layer and effectively suppress co-channel interference.

Upon the successful capture and demodulation of the data packet, the physical layer reports the actual signal-to-noise ratio (SNR) and RSSI of the link to the network protocol stack. Consequently, this paper defines the physical constraint for successful packet delivery as follows: predicated on fulfilling the aforementioned capture effect, the effective SNR of the target packet must be no less than the limit demodulation threshold $\gamma_{\mathrm{th}}\left(\mathrm{SF}\right)$ corresponding to the current SF. In summary, dense concurrent co-channel collisions constitute the fundamental physical cause for the severe performance degradation in high-load networks, simultaneously imposing stringent cross-layer collaborative design requirements on the collision isolation capability at the MAC layer and the routing link selection at the network layer.

## 3. Cross-Layer Distributed LoRa Ad Hoc Network Protocol Design

### 3.1. Optimizing Relay Deployment in Hybrid Topologies

To address the building obstructions and communication dead zones described in Section 2, traditional LoRaWAN star topologies fail to provide full-area coverage due to the lack of routing capabilities. Accordingly, this subsection proposes a distributed relay mechanism based on a layered “star–mesh” hybrid self-organizing network topology to dynamically expand network coverage.

#### 3.1.1. Dynamic Classification of End Nodes

The system employs a distributed dynamic relay strategy, dynamically classifying end nodes based on the link margin and providing on-demand services. Let the initial coverage area of the gateway be denoted as *C*. Based on the demodulation thresholds of the orthogonal spreading factors, the theoretical LOS coverage range of the gateway can be partitioned into multiple annular regions. However, in practical complex environments, subjected to the combined effects of building obstruction, multi-path effects, and instantaneous co-channel interference, the physical link quality exhibits time-varying characteristics and uncertainty. End nodes originally located within the theoretical coverage range of the gateway may also suffer from link disruptions due to sudden environmental fading. In practical LoRa communications, the received signal power equals the RSSI only for positive values of the SNR; when the system operates under negative SNR conditions, the actual receiver power is the sum of the RSSI and the SNR. To more accurately evaluate the true state of the underlying physical link, the relay node eavesdrops on the uplink packets transmitted by the end node and calculates the uplink link margin $M_{{\mathrm{SF}}_{i}}$ from the end node to the gateway using a piecewise function:

(6) $$M_{{\mathrm{SF}}_{i}}=\left\{\begin{array}{cc} {\mathrm{RSSI}}_{{\mathrm{SF}}_{i}}-P_{\mathrm{sens}}-\delta_{\mathrm{SF}}, & \mathrm{if}\;\mathrm{SNR}\geq 0\\ \left({\mathrm{RSSI}}_{{\mathrm{SF}}_{i}}+\mathrm{SNR}\right)-P_{\mathrm{sens}}-\delta_{\mathrm{SF}}, & \mathrm{if}\;\mathrm{SNR}<0 \end{array}\right.$$

where ${\mathrm{RSSI}}_{{\mathrm{SF}}_{i}}$ is the estimated received signal strength at the gateway, $P_{\mathrm{sens}}$ is the hardware receiver sensitivity of the gateway under the corresponding spreading factor, and $\delta_{{\mathrm{SF}}_{i}}$ is the uncertainty fading margin. Based on the link margin evaluation results, the network dynamically classifies the end nodes into two categories:

- Directly reachable end nodes (reachable ENs): If $M_{{\mathrm{SF}}_{i}}\geq 0$, it indicates that the current link condition is favorable, enabling single-hop communication with the gateway via the standard star topology. To conserve spectrum and energy, the relay nodes remain silent upon receiving data packets from such end nodes.
- Unreachable end nodes (unreachable ENs): If $M_{{\mathrm{SF}}_{i}}<0$, it indicates that the direct links of such nodes may have failed due to long distances or other factors, necessitating multi-hop forwarding via relay nodes. Upon receiving the routing requests and data packets from these end nodes, the relay nodes forward them to the gateway via a mesh backbone link.

**Figure 6:**
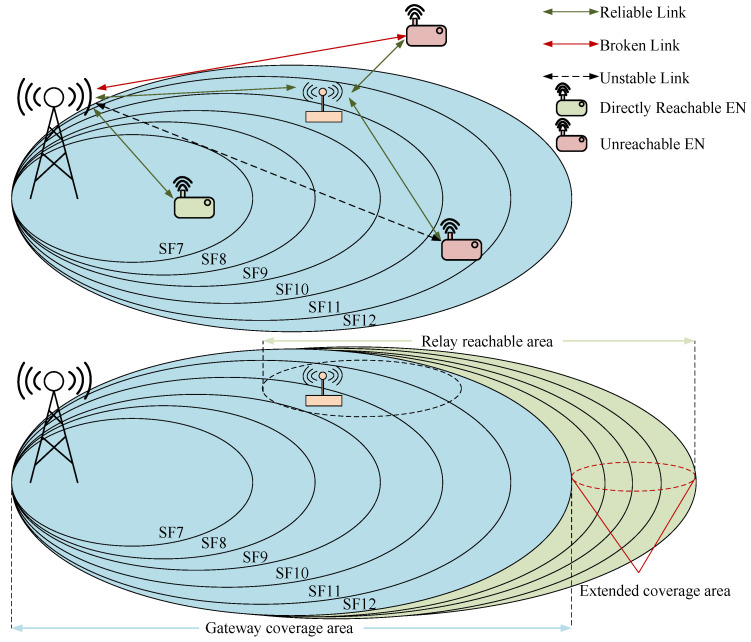
Topology of dynamic classification of end nodes and coverage extension.

Based on the aforementioned link margin evaluation and end node classification mechanisms, the extended coverage topology of the distributed dynamic relay hybrid network is illustrated in Figure 6.

#### 3.1.2. Relay Deployment Optimization Model

The spatial deployment position $Z_{r}$ of the relay nodes directly determines the coverage enhancement effect of the hybrid topology on the unreachable end nodes in blind spots. An ideal deployment position must simultaneously satisfy two conditions: it must effectively receive the weak signals from the nodes in the blind spots, and its own backhaul link to the gateway must possess a stable and reliable SNR and RSSI [5].

Let the set of candidate relay locations be denoted as *Z*, and the set of unreachable end nodes as $D_{\mathrm{unreach}}$. Define a Boolean coverage variable $C\left(i,Z_{r}\right)\in \left\{0,1\right\}$, where $C(i,Z_{r})=1$ if the relay node deployed at $Z_{r}$ satisfies the demodulation threshold and successfully receives the signal from end node *i*. The relay deployment optimization problem can be formulated as maximizing the coverage utility function:

(7) $$\max_{Z_{r}\in Z}\sum_{i\in D_{\mathrm{unreach}}}C\left(i,Z_{r}\right)\cdot P_{\mathrm{succ}}^{r}\left(d_{i,r}\right)$$

This optimization objective is subject to the following constraints:

(8) $$s.t.\;\left\{\begin{array}{c} C_{1}:\mathrm{SNR}\left(Z_{r}\to \mathrm{GW}\right)\geq \gamma_{\mathrm{th}}\left({\mathrm{SF}}_{\mathrm{backhaul}}\right)\\ C_{2}:\mathrm{RSSI}\left(Z_{r}\to \mathrm{GW}\right)\geq P_{\mathrm{sens}}\\ C_{3}:\Omega \left(Z_{r}\right)\leq \left\lfloor \frac{\alpha_{r}\cdot T}{T_{\mathrm{ToA},\mathrm{SF}}}\right\rfloor \\ C_{4}:P_{\mathrm{tx},r}\leq P_{\max} \end{array}\right.$$

where $d_{i,r}$ is the distance between the unreachable end node *i* and the relay node *r*; $P_{\mathrm{succ}}^{r}\left(d_{i,r}\right)$ is the expectation of successful physical link delivery at distance $d_{i,r}$; constraints $C_{1}$ and $C_{2}$ represent the backhaul link reliability constraints, which require that the link from the relay node deployed at position $Z_{r}$ to the gateway must satisfy the minimum SNR demodulation threshold and the hardware sensitivity limit $P_{\mathrm{sens}}$ corresponding to the backhaul spreading factor; constraint $C_{3}$ is the relay capacity constraint, wherein the relay node must comply with the duty cycle regulatory limit $\alpha_{r}$ of the LoRa band, and its actual packet forwarding load $\Omega (Z_{r})$ to be carried within period *T* is restricted by the time-on-air ($T_{\mathrm{ToA},\mathrm{SF}}$); and constraint $C_{4}$ is the maximum transmit power constraint of the ISM band.

#### 3.1.3. Relay Deployment Optimization Algorithm

Addressing the relay deployment optimization problem, the severe NLOS fading in 3D space and multiple non-linear constraints render it a multi-modal, non-convex NP-hard problem. Traditional greedy strategies are highly prone to getting trapped in local optima within complex environments, whereas purely heuristic random searches suffer from slow convergence. To address this, in the context of complex scenarios constructed using 3D bounding boxes, this paper proposes a two-stage hybrid heuristic optimization algorithm. By combining the greedy set cover approach with simulated annealing (SA), it jointly solves for the 3D deployment positions of the relay nodes.

The core procedure of this hybrid algorithm consists of two stages:

Stage 1: Discretized initial deployment based on a greedy strategy.

To rapidly obtain a high-quality initial feasible solution, the continuous 3D deployment space is first discretized into a set of grid candidate points with a fixed step size. By integrating the spatial model with the link margin evaluation mechanism, the uplink communication quality from the end nodes to the gateway is exhaustively evaluated to identify the unreachable end nodes. Subsequently, a greedy algorithm is employed. In each iteration, priority is given to deploying a relay at the grid point that can cover the maximum number of end nodes in blind spots while satisfying the backhaul SNR and RSSI constraints (constraints $C_{1},C_{2}$) and the maximum transmit power limit (constraint $C_{4}$). During the coverage access process, the system strictly enforces admission control. Once the traffic load carried by the relay reaches the capacity upper bound stipulated by the LoRa duty cycle regulations (constraint $C_{3}$), the remaining end nodes in the blind spots are denied access and deferred to subsequent iterations to be covered by newly deployed relays. This process continues until all end nodes in the blind spots are completely covered. This stage rapidly generates an initial relay set $R_{\mathrm{init}}$ with complete connectivity.

Stage 2: Refined optimization in 3D continuous space based on simulated annealing.

Taking $R_{\mathrm{init}}$ from Stage 1 as the initial state, the simulated annealing (SA) algorithm is employed to perform refined position adjustments within the 3D continuous space.

1. Neighborhood perturbation: At the current temperature *T*, a dynamic Gaussian perturbation is applied to the 3D coordinates (x,y,z) of the relay node to generate a neighborhood solution.
2. Cost evaluation and penalty mechanism: The system cost function Cost aims to minimize the penalty for uncovered end nodes and the cost associated with violating the backhaul constraints. Let the basic coverage utility be denoted as U(R), which represents the sum of the joint coverage utilities of all coordinate points within the currently deployed relay position set *R*. It is defined as the spatial summation of the single-node objective function from Section 3.1.2 over the position set *R*:
  (9) $$U\left(R\right)=\sum_{Z_{r}\in R}\left(\sum_{i\in D_{\mathrm{unreach}}}C\left(i,Z_{r}\right)\cdot P_{\mathrm{succ}}^{r}\left(d_{i,r}\right)\right)$$
  To transform the constrained deployment problem into an unconstrained optimization problem, the system cost function of the algorithm is defined as follows:
  (10) $$\mathrm{Cost}=-U\left(R\right)+\omega \cdot \sum_{Z_{r}\in R}\mathrm{Penalty}\left(Z_{r}\right)$$
  where ω is a sufficiently large penalty weight constant; $\mathrm{Penalty}(Z_{r})$ is the constraint penalty term, which equals 1 if the newly deployed relay position $Z_{r}$ violates the backhaul SNR/RSSI constraints (constraints $C_{1},C_{2}$) or if the end node load carried by the relay exceeds the duty cycle capacity limit (constraint $C_{3}$), and 0 otherwise.
3. Metropolis criterion: If the system cost of the new position decreases, the new solution is accepted directly; if the cost increases, this inferior solution is accepted with a certain probability, thereby empowering the algorithm to escape from local optima. As the temperature gradually decreases according to the cooling rate, the algorithm eventually converges to a near-global optimal deployment position.

The detailed steps are outlined as follows (Algorithm 1):
**Algorithm 1:** Relay Deployment Optimization Based on a Hybrid Greedy and Simulated Annealing Strategy
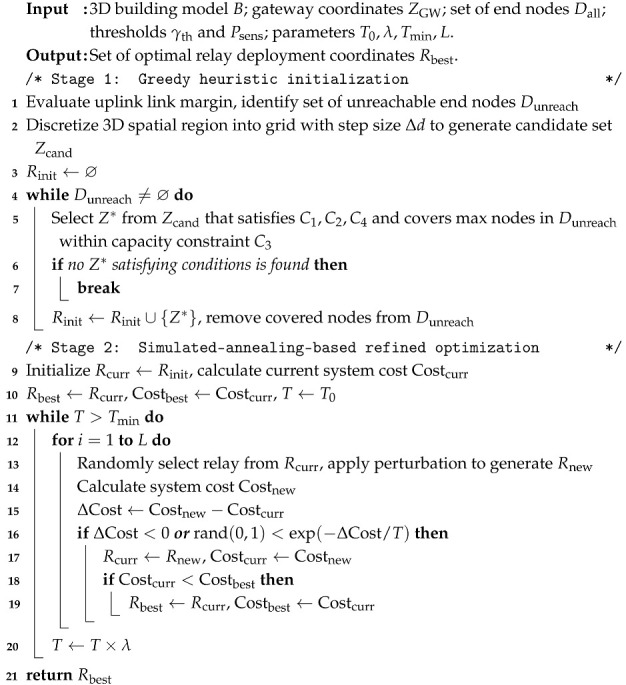


### 3.2. Priority-Based MAC Access Mechanism

#### 3.2.1. Heterogeneous Traffic Characteristics

The network accommodates three types of traffic with distinct QoS constraints. Specifically, periodic data mainly comprises periodic environmental sensing information, serving as the fundamental network load. It features a fixed generation frequency and a relatively large data volume, and can tolerate a certain degree of queuing congestion and transmission delay. Emergency data is triggered by abnormal events at the end nodes. Being delay-sensitive, it must be assigned the highest non-preemptive scheduling priority to minimize the end-to-end uplink delay. Control commands are action directives issued by the gateway to the end nodes. Although their downlink data volume is small, they demand high transmission reliability, requiring accurate and lossless delivery in complex obstructed environments.

#### 3.2.2. Non-Preemptive Priority Queuing Model

**Figure 7:**
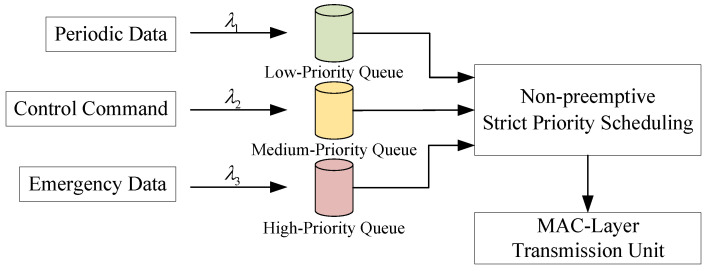
Multi-priority queuing scheduling architecture.

To formally describe the buffering and scheduling process of heterogeneous traffic at the nodes, we adopt a non-preemptive priority queuing model. Considering the previously discussed traffic characteristics, Figure 7 illustrates the multi-priority queuing and scheduling architecture.

It is assumed that the arrival processes of the traffic packets for each priority, whether generated or forwarded by the nodes, are mutually independent and all follow a Poisson process with parameter $\lambda_{k}\;\left(k\in \left\{1,2,3\right\}\right)$ [18]. As illustrated in Figure 7, three mutually independent buffer queues are logically maintained within the node. According to the QoS requirements, emergency data with an arrival rate of $\lambda_{3}$ is mapped to the high-priority queue [10], control commands with an arrival rate of $\lambda_{2}$ are mapped to the medium-priority queue, and periodic data with an arrival rate of $\lambda_{1}$ is mapped to the low-priority queue.

During the packet forwarding phase, the MAC layer transmission unit acts as a single server, whose service time follows a general distribution with a mean of $1/\mu_{k}$. When the transmission unit is idle, the non-preemptive priority scheduler adopts a strict priority scheduling policy: it sequentially extracts packets from the medium- and low-priority queues for transmission only when the high-priority queue is empty.

As the modulation characteristics of the LoRa physical layer preclude lossless interruption during transmission, forcibly truncating the ongoing chirp signal would lead to packet corruption and energy loss. Consequently, a non-preemptive scheduling mechanism is adopted in this paper. Once the transmission of a low-priority packet commences, even if a high-priority emergency packet arrives in the interim, the scheduler will wait for the current transmission to complete before prioritizing this emergency data in the subsequent scheduling cycle.

**Figure 8:**
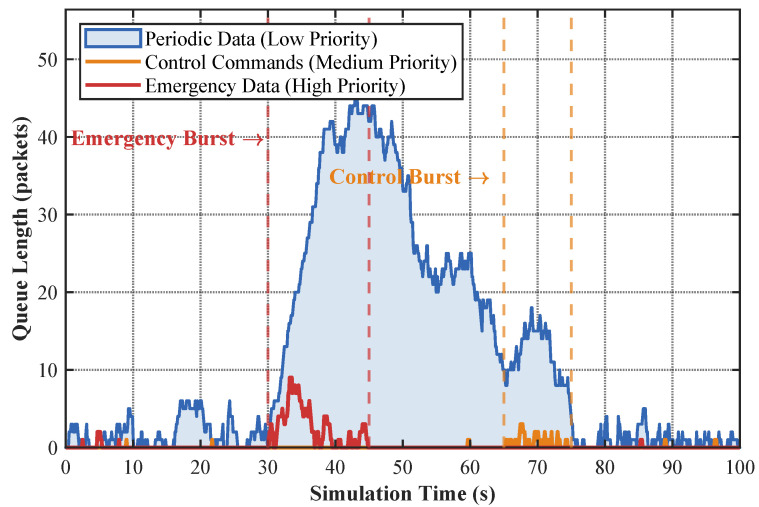
Dynamic evolution of three-level queue lengths.

As illustrated in Figure 8, with the introduction of the burst traffic model, it is evident that during the stable operation phase of the network, the queue backlog lengths of all three types of traffic remain at extremely low levels. However, when the network encounters a sudden emergency alarm event at 30 s, the channel transmission resources are rapidly taken over by the high-priority traffic. Consequently, the low-priority periodic data queue is effectively suppressed, resulting in a substantial buffer backlog (indicated by the peak in the light blue area). Meanwhile, although the high-priority data experiences a brief backlog during the burst period, it demonstrates significant scheduling advantages over the low-priority data in terms of both the backlog severity and the dissipation rate. Similarly, when medium-priority control commands are issued, they also exhibit effective suppression over the regular traffic.

#### 3.2.3. Differentiated Channel Contention and Backoff Mechanism

After being filtered and scheduled by the internal priority queues of the node, the data packets must directly access the congested and dynamically changing common wireless channel when delivered to the lower layer. To prevent concurrent collisions caused by the simultaneous access of a large number of nodes, and to address the blind access issue inherent in the traditional LoRaWAN pure ALOHA mechanism, this mechanism introduces a differentiated backoff algorithm based on the synergy of symbol energy detection and contention window (CW) [7,8], thereby enhancing the utilization of air-interface resources.

**Figure 9:**
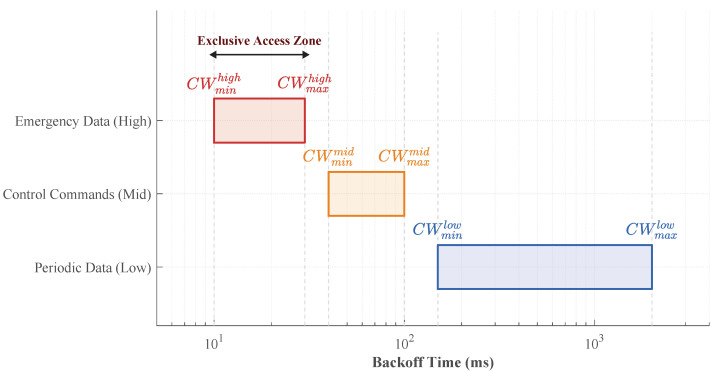
Time-domain isolation and backoff distribution of differentiated contention windows.

1. Channel Sensing and Collision Avoidance Based on Symbol Energy Detection
  Before initiating data transmission, the transmitting node must execute the CAD mechanism to assess the idle state of the target channel. Given the physical characteristics of LoRa’s linear CSS modulation, the energy of a single LoRa symbol is spread uniformly across the entire channel bandwidth. The CAD mechanism achieves precise detection of specific symbol energy by performing coherent correlation matching between a locally generated standard chirp waveform and the received signal. This process effectively identifies valid LoRa symbols operating with the same channel BW and SF. To realize adaptive channel sensing, the system dynamically adjusts the CAD correlation peak decision threshold by incorporating the environmental noise floor. If the correlation peak exceeds the predefined threshold, it indicates the presence of an LoRa signal with identical parameters, rendering the channel busy; consequently, the node executes a random backoff and defers its current channel access. Conversely, if no matching chirp is detected, the channel is deemed idle, granting the node permission to initiate transmission. This chirp-feature-aware LBT mechanism effectively mitigates packet collisions caused by concurrent transmissions from nodes sharing the same spreading parameters.
2. Traffic-aware differentiated backoff window
  When the channel transitions from a busy to an idle state, or when multiple concurrent nodes simultaneously complete their sensing and prepare to seize the channel, secondary collisions are highly prone to occur. To ensure that high-priority traffic secures prioritized access opportunities during channel contention, the MAC layer configures non-overlapping backoff window intervals for packets in different queues. This achieves a strict preemptive advantage through absolute isolation on the time axis. Specifically, a minimal CW of $\left[0,CW_{\max}^{\mathrm{high}}\right]$ is allocated to the high-priority burst emergency data; a medium CW of $\left[CW_{\min}^{\mathrm{mid}},CW_{\max}^{\mathrm{mid}}\right]$ is assigned to the medium-priority control commands; and a CW with an extremely large span of $\left[CW_{\min}^{\mathrm{low}},CW_{\max}^{\mathrm{low}}\right]$ is allocated to the low-priority periodic data. The probabilistic isolation characteristics of the differentiated CWs for the aforementioned three types of heterogeneous traffic in the time domain are illustrated in Figure 9.

Through strict parameter configuration, the system ensures that the upper and lower bounds of the backoff intervals satisfy $CW_{\max}^{\mathrm{high}}<CW_{\min}^{\mathrm{mid}}$ and $CW_{\max}^{\mathrm{mid}}<CW_{\min}^{\mathrm{low}}$, thereby establishing a dedicated access interval isolated in the time domain for emergency data. Upon channel release, emergency nodes require only a minimal random delay to initiate packet transmission first. This completely eliminates the contention eligibility of the other two types of traffic, granting emergency data the highest channel preemption success rate. Meanwhile, medium-priority signaling proactively avoids emergency data in the time domain, whereas the massive volume of regular periodic data is forced to back off within an extremely broad dispersion interval.

### 3.3. CAM-AODV Routing Algorithm

Traditional routing protocols typically rely on the minimum hop count as the sole metric for path selection. In complex obstructed and high-density access scenarios, they are prone to selecting long links with insufficient communication margins. Moreover, the lack of load and energy awareness exacerbates network congestion and curtails node lifespan, making it difficult to guarantee network stability [30,31]. To address this issue, this paper proposes a cross-layer multi-metric routing algorithm. By comprehensively sensing the hop count, link quality, queue status, and residual node energy, the proposed algorithm enhances transmission reliability and prolongs the network lifetime.

#### 3.3.1. Routing Metric Factor

To achieve highly reliable and long-lasting data transmission, the proposed algorithm extracts and calculates the following four core routing metric factors in a cross-layer manner during the route discovery phase:

1. Hop count metric.
  The hop count intuitively reflects the topological length of a routing path. A smaller hop count typically implies fewer forwarding nodes and lower cumulative delay. To ensure rapid convergence of the routing algorithm and to effectively prevent routing loops, the network assigns a fixed fundamental cost $C_{\mathrm{hop}}$ to each multi-hop forwarding event. This ensures that when other network states are similar, traffic is preferentially transmitted along the topologically shortest paths.
2. Link reliability.
  RSSI can effectively evaluate the reliability of the underlying physical link during data transmission [14]. Although the physical layer has performed hard-decision demodulation based on the SNR, the network layer still needs to further extract continuous signal strength features to evaluate the fading margin of the link. In complex obstructed environments, if a node with a weak received signal is selected as the next hop, slight environmental perturbations can easily trigger a link outage.
  Consequently, this paper extracts the RSSI value from the underlying physical module in a cross-layer manner, and configures the RSSI threshold $T_{\mathrm{RSSI}}$ by incorporating the underlying RF characteristics of the LoRa hardware. According to spread spectrum communication theory, this threshold is defined as the sum of the receiver sensitivity floor and the link fading margin:
  (11) $$T_{\mathrm{RSSI}}=-174+10\log_{10}\left(\mathrm{BW}\right)+\mathrm{NF}+{\mathrm{SNR}}_{\mathrm{limit}}\left(\mathrm{SF}\right)+P_{\mathrm{margin}}$$
  where −174dBm/Hz is the thermal noise power spectral density at room temperature; BW is the signal bandwidth; NF is the noise figure of the receiver hardware; ${\mathrm{SNR}}_{\mathrm{limit}}\left(\mathrm{SF}\right)$ is the minimum signal-to-noise ratio required for successful demodulation under a specific spreading factor; and $P_{\mathrm{margin}}$ is the shadow fading margin introduced to cope with complex obstructed environments.
  When the link RSSI is lower than $\mathrm{RSSI}<T_{\mathrm{RSSI}}$, the link is deemed unreliable and is discarded. For valid links, considering that lower signal strength exponentially increases the bit error rate (BER), the link fading cost is defined as being proportional to the absolute value of the RSSI. To simplify the computation, this paper employs a linear mapping of the absolute RSSI value; the link quality cost from node *i* to node *j* is modeled as follows:
  (12) $$C_{\mathrm{link}}\left(i,j\right)=\frac{|{\mathrm{RSSI}}_{i,j}|}{|T_{\mathrm{RSSI}}|}$$
3. Congestion metric.
  Congestion significantly increases network latency and packet loss rates, degrades throughput, and exacerbates additional energy consumption overhead caused by collision-induced retransmissions. To quantify the congestion level of a node, the current node *j* dynamically senses the queue backlog length $Q_{\mathrm{len}}$ in its MAC layer buffer, which characterizes the queue occupancy rate of the link over a specific time period. A higher queue occupancy rate implies a greater potential for queuing delay and collision risk. Let $Q_{\max}$ be the maximum packet capacity of node *j*; its congestion load cost is defined as follows:
  (13) $$C_{\mathrm{cong}}\left(j\right)=\frac{Q_{\mathrm{len}}\left(j\right)}{Q_{\max}}$$
4. Residual energy.
  Most traditional routing strategies rely solely on hop count, neglecting the issue of uneven node energy dissipation. Frequently selecting the same shortest path for data transmission can easily lead to the rapid depletion of energy in critical nodes, causing local topology fragmentation and network partitioning. To extend the overall network lifetime, this paper introduces a non-linear residual energy penalty model. Based on the energy consumption model in Section 2.2, the system calculates the residual energy ratio of node j in real time and imposes a quadratically increasing survival penalty, compelling traffic to actively bypass near-depleted nodes:
  (14) $$C_{\mathrm{energy}}\left(j\right)={\left(1-\frac{E_{\mathrm{res}}\left(j\right)}{E_{\mathrm{init}}\left(j\right)}\right)}^{2}$$
  where $E_{\mathrm{res}}\left(j\right)$ and $E_{\mathrm{init}}\left(j\right)$ denote the current residual energy and the initial energy of the node, respectively.

#### 3.3.2. Differentiated Routing Cost Function

In conjunction with the previously designed MAC layer non-preemptive priority scheduling mechanism, the underlying routing must align with the QoS requirements of the upper-layer traffic. To this end, this paper proposes a differentiated cost function model based on traffic characteristics and introduces a dynamic weight adaptation mechanism to cope with complex and time-varying network loads.

1. Global balanced routing model.
  Periodic data exhibits a certain degree of delay tolerance. Thus, the routing strategy takes global load balancing and energy distribution as its core objectives, comprehensively computing all four metric factors during route discovery. The comprehensive link cost from node i to node j is defined as follows:
  (15) $${\mathrm{Cost}}_{\mathrm{low}}\left(i,j\right)=C_{\mathrm{hop}}+\alpha \cdot C_{\mathrm{link}}\left(i,j\right)+\beta \cdot C_{\mathrm{cong}}\left(j\right)+\gamma \cdot C_{\mathrm{energy}}\left(j\right)$$
  where ${\mathrm{Cost}}_{\mathrm{low}}\left(i,j\right)$ is the comprehensive link cost calculated for low-priority periodic data; α,β,γ∈(0,1) and α+β+γ=1. This model effectively avoids the issues of congestion and node energy depletion caused by traditional shortest-path routing.
2. Asymmetric routing cost model.
  For urgent data and control signaling, the core requirements are low latency and high connectivity. The routing layer employs an asymmetric metric strategy for such traffic; during link cost computation, it exempts the congestion and energy consumption penalties, solely selecting the optimal path with the minimum physical fading and the shortest hop count. Its cost function reduces to
  (16) $${\mathrm{Cost}}_{\mathrm{urgent}}\left(i,j\right)=C_{\mathrm{hop}}+C_{\mathrm{link}}\left(i,j\right)$$
  where ${\mathrm{Cost}}_{\mathrm{urgent}}\left(i,j\right)$ is the asymmetric link cost calculated for medium- and high-priority traffic. By eliminating secondary constraints, this model enhances the end-to-end delivery efficiency of medium- and high-priority data.

#### 3.3.3. Dynamic Weight Adaptation Mechanism

In practical applications, the two aforementioned cost models face challenges posed by the burstiness and time-varying nature of network loads. If the weight coefficients (α,β,γ) in the cost functions are statically configured, the network may fall into local sub-optima under specific scenarios. To address this, this paper proposes a dynamic weight adaptation closed-loop mechanism based on macroscopic network state feedback. In this mechanism, the gateway periodically evaluates the global network state and updates the parameters accordingly.

Let Δt denote the state evaluation update period of the system. During the k-th observation period, the global PDR aggregated by the gateway is denoted as PDR(k), and the network-wide average end-to-end delay is denoted as Delay(k). The system defines a reliability safety threshold range $\left[{\mathrm{PDR}}_{\mathrm{th}\_\mathrm{low}},{\mathrm{PDR}}_{\mathrm{th}\_\mathrm{high}}\right]$ and a delay tolerance threshold range $\left[{\mathrm{Delay}}_{\mathrm{th}\_\mathrm{low}},{\mathrm{Delay}}_{\mathrm{th}\_\mathrm{high}}\right]$. The specific weight adjustment mechanism is as follows:

1. Link quality weight adaptation.
  When $\mathrm{PDR}\left(k\right)<{\mathrm{PDR}}_{\mathrm{th}\_\mathrm{low}}$, it indicates severe physical fading or collisions in the network. The system must increase α to guide routing decisions to converge towards high signal-to-noise ratio (SNR) links. Conversely, if the network state is stable with $\mathrm{PDR}\left(k\right)>{\mathrm{PDR}}_{\mathrm{th}\_\mathrm{high}}$, the physical layer constraints are appropriately relaxed, and this weight is gradually reduced. The complete update equation is defined as follows:
  (17) $$\alpha \left(k+1\right)=\left\{\begin{array}{cc} \min(\alpha \left(k\right)\cdot \left(1+\mu_{1}\right),\alpha_{\max}), & \mathrm{PDR}\left(k\right)<{\mathrm{PDR}}_{\mathrm{th}\_\mathrm{low}}\\ \max(\alpha \left(k\right)\cdot \left(1-\mu_{2}\right),\alpha_{\min}), & \mathrm{PDR}\left(k\right)>{\mathrm{PDR}}_{\mathrm{th}\_\mathrm{high}}\\ \alpha (k), & \mathrm{otherwise} \end{array}\right.$$
  where $\mu_{1}$ and $\mu_{2}$ denote the smoothing step sizes; and $\alpha_{\max}$ and $\alpha_{\min}$ represent the permissible upper and lower bounds of the link weight, respectively.
2. Congestion load weight adaptation.
  When $\mathrm{Delay}\left(k\right)>{\mathrm{Delay}}_{\mathrm{th}\_\mathrm{high}}$, it indicates a high risk of queue overflow at the core relay nodes. The algorithm dynamically increases the congestion penalty coefficient β, guiding subsequent concurrent traffic to be diverted towards idle edge relay nodes. Conversely, if the network delay falls below the lower tolerance limit ${\mathrm{Delay}}_{\mathrm{th}\_\mathrm{low}}$, this coefficient is gradually reduced. The update equation for the congestion load weight is as follows:
  (18) $$\beta \left(k+1\right)=\left\{\begin{array}{cc} \min(\beta \left(k\right)+\Delta \beta ,\beta_{\max}), & \mathrm{Delay}\left(k\right)>{\mathrm{Delay}}_{\mathrm{th}\_\mathrm{high}}\\ \max(\beta \left(k\right)-\Delta \beta ,\beta_{\min}), & \mathrm{Delay}\left(k\right)<{\mathrm{Delay}}_{\mathrm{th}\_\mathrm{low}}\\ \beta (k), & \mathrm{otherwise} \end{array}\right.$$
  where Δβ denotes the linear adjustment step size of the congestion weight; and $\beta_{\max}$ and $\beta_{\min}$ represent the permissible upper and lower bounds of the congestion weight, respectively.
3. Residual energy weight adaptation.
  To ensure the mathematical rigor of the multi-objective linear weighted decision making, the system weights must satisfy the normalization constraint α+β+γ=1. After completing the independent updates of α and β, the system adaptively calculates the residual energy weight γ based on the normalization condition:
  (19) γ(k+1)=1−α(k+1)−β(k+1)
  To prevent the scenario where α and β simultaneously reach their peak values under extreme conditions characterized by concurrent link degradation and network congestion, which would cause γ to become non-positive, the system enforces a boundary constraint of $\alpha_{\max}+\beta_{\max}<1$ during parameter initialization. This constraint ensures that γ(k+1) strictly remains positive. Consequently, under any time-varying network load, the algorithm consistently retains its fundamental protection capability for low-energy nodes.

By introducing the aforementioned closed-loop mechanism, the weight parameters in the algorithm can be adjusted in real time in response to fluctuations in the network-wide PDR and delay, thereby ensuring the robustness of the differentiated routing strategy in complex environments.

#### 3.3.4. Implementation of the Improved Routing Algorithm

To support cross-layer multi-metric computation and differentiated routing for heterogeneous traffic, this paper reconstructs the routing control packet (RREQ/RREP) structures and the routing table interaction logic of the traditional AODV protocol. The enhanced routing mechanism overcomes the limitation of lacking global awareness inherent in the first-come-first-served route establishment strategy of the traditional protocol, employing cost-optimization-based delayed decision making and data-driven soft-state management.

**Figure 10:**
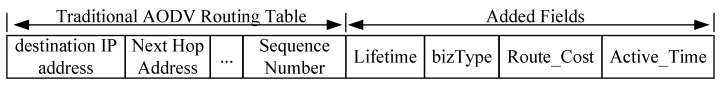
Extended routing table structure of the CAM-AODV protocol.

**Figure 11:**
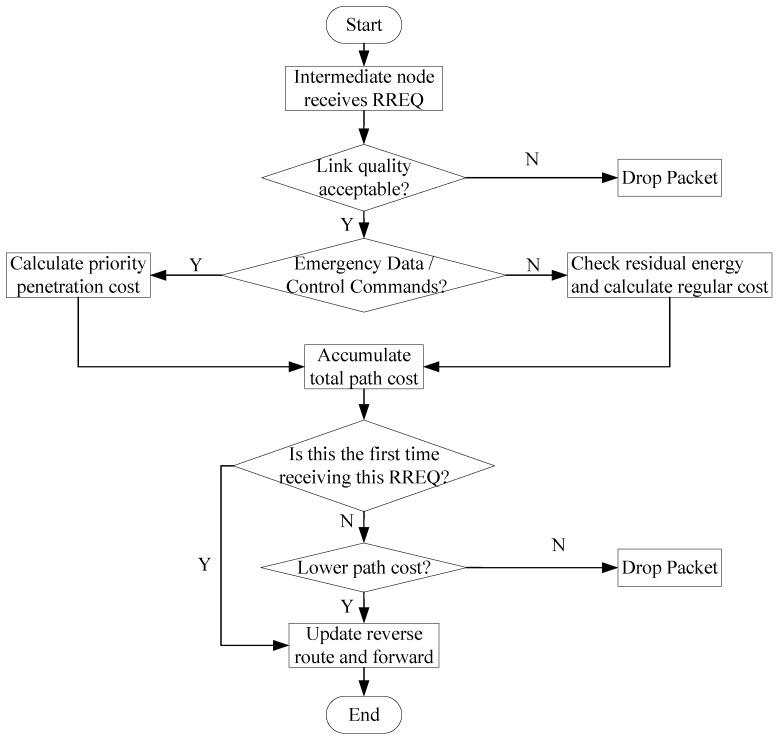
Flowchart of cross-layer multi-metric route establishment.

**Figure 12:**
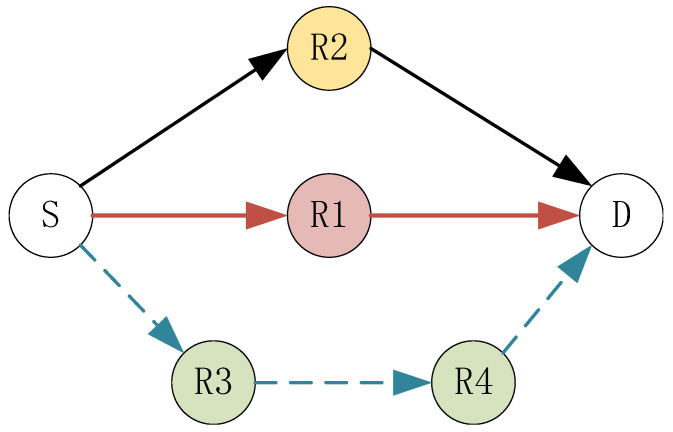
Multi-metric routing steering decisions under heterogeneous traffic.

**Figure 13:**
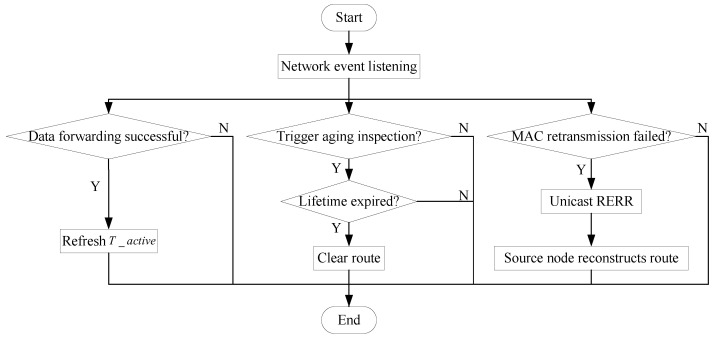
Flowchart of the event-driven route maintenance mechanism.

1. Enhancements to the routing table.
  During the route discovery phase, building upon the traditional fields, the RREQ packet broadcast by the source node incorporates two core fields: the service priority identifier (bizType) and the cumulative path cost (Path_Cost). During the route reply phase, as the destination node adopts a collection window-based delayed optimal decision-making mechanism, the returned unicast RREP packet also introduces the bizType field to explicitly indicate the specific service type served by this optimal path.
  Correspondingly, regarding the maintenance of the node’s local state, to achieve precise steering of heterogeneous traffic, the lookup criterion for the local routing table at network nodes is expanded from the traditional single destination address to a combination of the destination address and the service priority identifier (bizType). As the RREP returns hop-by-hop to the source node along the reverse link, intermediate nodes strictly parse the bizType field within the packet and, accordingly, establish independent forward routing entries for different service flows. Simultaneously, two new fields are added to the routing table entries: the comprehensive route cost (Route_Cost) and the active timestamp (Active_Timestamp). These fields not only provide a comparison baseline for the cost optimization of subsequent duplicate RREQs, but also serve as the basis for the soft-state-based route timeout and aging mechanism. The extended routing table structure of the enhanced CAM-AODV protocol is illustrated in Figure 10.
2. Route establishment process.
  When a source node initiates communication with a destination node, if no valid route exists locally, a distributed route discovery flooding is triggered. The specific algorithm is illustrated in Figure 11.
  Step 1: Upon receiving an RREQ packet, the intermediate node first retrieves the RSSI provided by the physical layer through cross-layer interaction. If $\mathrm{RSSI}<T_{\mathrm{RSSI}}$, the node determines that the link is on the verge of severe fading and directly drops the RREQ, thereby preventing the establishment of low-quality links from the outset. If the signal quality is acceptable, it further parses the bizType field within the packet to determine which cost function to employ subsequently.
  Step 2: Based on the determined cost function, the node calculates the comprehensive cost ΔCost of the current hop by integrating its current queue length and residual energy, and accumulates this value into the Path_Cost field of the RREQ. Subsequently, the node records the sending node of the RREQ as the next hop for the reverse route.
  Step 3: In traditional AODV, nodes directly drop all duplicate RREQs to avoid broadcast storms [17]. In contrast, in the proposed mechanism, to strike a balance between route optimization and signaling overhead, a cost improvement threshold $\Delta {\mathrm{Cost}}_{\mathrm{th}}$ is introduced. Upon receiving a duplicate RREQ with the same Broadcast ID, the intermediate node compares the Path_Cost in the current packet with the historical minimum cost Min_Cost. Only when $\mathrm{Min}\_\mathrm{Cost}-\mathrm{Path}\_\mathrm{Cost}>\Delta {\mathrm{Cost}}_{\mathrm{th}}$ does it indicate that a superior alternative path has been discovered. In this case, the node forcibly updates the reverse routing table and continues broadcasting the RREQ; otherwise, it drops the packet.
  Step 4: Upon receiving the first RREQ, the destination node does not reply immediately; instead, it opens a cost collection window with a duration of $T_{\mathrm{wait}}$. During this window, the destination node receives RREQs arriving via multiple different topological paths. Upon the expiration of the window, the destination node evaluates all collected candidate paths, selects the one with the minimum Path_Cost as the global optimal path, and unicasts a Route Reply (RREP) packet along the reverse route, enabling intermediate nodes along the path to establish the forward route.
  As illustrated in Figure 12, three candidate paths to the destination gateway D exist within the current local topology: Path A ($S\to R_{1}\to D$), which features the minimum number of physical hops (two hops) but suffers from severe MAC-layer queue congestion at relay node $R_{1}$; Path B ($S\to R_{2}\to D$), which also consists of two hops but experiences slightly inferior link quality, with the residual energy of its relay node $R_{2}$ dropping below the safety threshold; and Path C ($S\to R_{3}\to R_{4}\to D$), which is topologically longer (three hops) but passes through intermediate relay nodes $R_{3}$ and $R_{4}$ that possess idle queues and sufficient energy.
  In this scenario, the proposed algorithm achieves precise QoS-based traffic steering: regular periodic data is governed by the comprehensive cost penalty, proactively avoiding the high-risk nodes $R_{1}$ and $R_{2}$, and detouring to the global optimal path d, thereby realizing network-wide load balancing and node lifetime protection; in contrast, emergency data is forcibly exempted from congestion and energy consumption penalties, directly preempting Path A, which features the minimum physical hops.
3. Route maintenance mechanism.
  To address the issue that the optional periodic HELLO message broadcast mechanism used in traditional AODV route maintenance is highly prone to causing air-interface collisions and consuming limited channel resources, this paper designs an event-driven route maintenance mechanism, as illustrated in Figure 13. This mechanism consists of three parallel triggering logics. First, data-driven refresh relies on the successful forwarding of service data by a node to automatically update the survival timestamp of the corresponding routing entry, thereby achieving passive route keep-alive. Second, timeout-based silent aging conducts periodic system inspections, directly marking paths that remain idle beyond a predefined threshold as invalid and clearing them to release the limited cache of the microcontroller. Third, cross-layer proactive self-healing occurs when the MAC layer reaches the maximum retransmission limit and detects a physical link breakage, prompting the network layer to unicast an RERR (Route Error) packet to notify the source node to reinitiate route discovery. By leveraging these mechanisms, the proposed algorithm significantly reduces the interference of redundant control signaling on data transmission, effectively improving the overall network PDR and channel utilization.

## 4. Simulation Evaluation and Result Analysis

### 4.1. Simulation Parameter Settings

To comprehensively evaluate the overall performance of the proposed hybrid topology relay deployment, priority-based MAC access, and the CAM-AODV cross-layer multi-metric routing protocol, this study independently developed a discrete-event-driven 3D hierarchical LoRa network simulation environment based on the MATLAB platform.

The simulation scenario is configured as a 3D space measuring 923 m × 498 m × 25 m, encompassing complex building clusters geometrically modeled using 3D Axis-Aligned Bounding Boxes (AABBs) to realistically replicate NLOS blockages and signal spatial penetration fading characteristics. Regarding node deployment, the gateway node is fixedly deployed on the rooftop of the core building; end nodes are randomly scattered within the target area, with their number increasing from 50 to 300; meanwhile, relay nodes are optimally and dynamically deployed using the hybrid simulated annealing algorithm proposed in Section 3.1. The specific macroscopic simulation parameters for the global network and physical layer are detailed in Table 2. At the physical layer parameter configuration, to ensure data transmission reliability and error-correction capability, the underlying packet coding rate (CR) for all nodes across the network is uniformly fixed at 4/5. For the assignment of SFs, the system relies on the uplink link margin piecewise function defined in Section 3.1.1. During the terminal initialization and relay deployment phases, the system adaptively assigns the minimum available SF between SF7 and SF12 based on the distance and fading conditions from the node to the gateway or relay. This strategy achieves an optimal trade-off between coverage range and $T_{\mathrm{ToA}}$.

Furthermore, to simulate the complex characteristics of heterogeneous traffic in real-world scenarios and to verify the non-preemptive priority queuing model and differentiated backoff mechanism proposed in Section 3.2, this simulation conducts a fine-grained classification of the traffic types for both end nodes and gateways. Based on the delay and reliability tolerance of the traffic, the network traffic is categorized into three major classes: emergency alarm traffic, control signaling traffic, and regular periodic traffic. The system allocates distinct payload sizes and backoff CWs to traffic of different priorities. The specific heterogeneous traffic types and scheduling parameter settings are detailed in Table 3.

To ensure the statistical significance and objectivity of the experimental results, the simulation evaluation circumvents the incidental errors potentially introduced by a single specific topology. For the subsequent evaluation scenarios involving varying network densities and loads, 20 independent random simulations were executed in MATLAB. In each independent run, the spatial distribution of the end nodes and the packet generation timestamps for the heterogeneous traffic were randomly regenerated. Consequently, the core performance metrics presented in Section 4.2, including the PDR, system throughput, and average end-to-end delay, are all statistical averages derived from these 20 independent simulation runs.

### 4.2. Analysis of Simulation Results

#### 4.2.1. Relay Deployment and Coverage Verification in a 3D Environment

**Figure 14:**
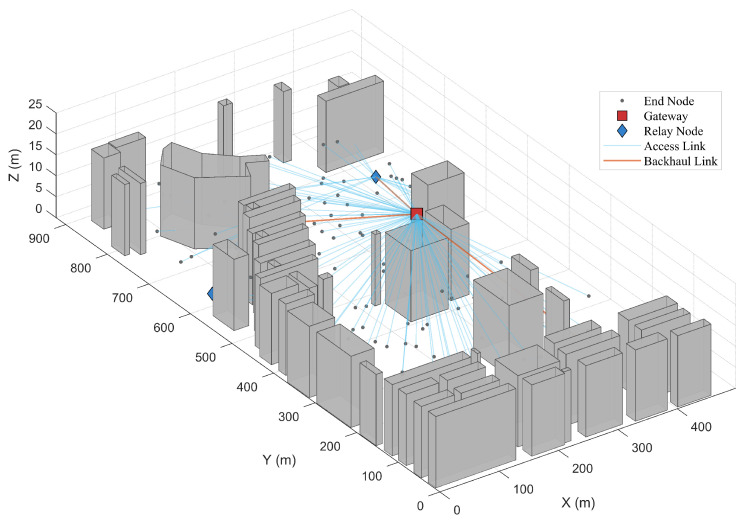
Ad hoc network topology and routing links based on the hybrid SA algorithm.

Based on the proposed hybrid simulated annealing relay deployment algorithm, a 3D topology was constructed in a scenario with 150 randomly distributed end nodes. As illustrated in Figure 14, the gateway (red square) is deployed on the rooftop of the core building, while the end nodes (gray dots) suffer from partial communication blind zones due to building blockages. The relay nodes (blue diamonds) selected by the algorithm establish connected backbone backhaul links (solid orange lines) within the 3D space.

Simulation results demonstrate that in a 3D building-dense scenario, the single-gateway direct-connectivity mode suffers from conspicuous communication coverage blind zones due to signal penetration loss and LOS blockage effects. By introducing the proposed hybrid SA relay optimization deployment algorithm, full coverage of all nodes across the entire network is achieved. Statistical analysis of the generated 3D routing topology reveals that the network-wide communication links exhibit a hybrid topological characteristic dominated by single-hop direct connections and supplemented by local multi-hop forwarding. This characteristic reflects the actual operational logic of the on-demand relay mechanism: end nodes within a favorable LOS coverage range preferentially adopt single-hop direct connections to minimize air-interface resource utilization; conversely, constrained end nodes located in NLOS or deep fading regions are allocated to the mesh backbone links for multi-hop forwarding. While compensating for physical coverage blind zones, this hierarchical hybrid mechanism effectively balances the spectrum efficiency and end-to-end transmission delay of the overall network.

#### 4.2.2. Impact of Network Density on Protocol Performance

**Figure 15:**
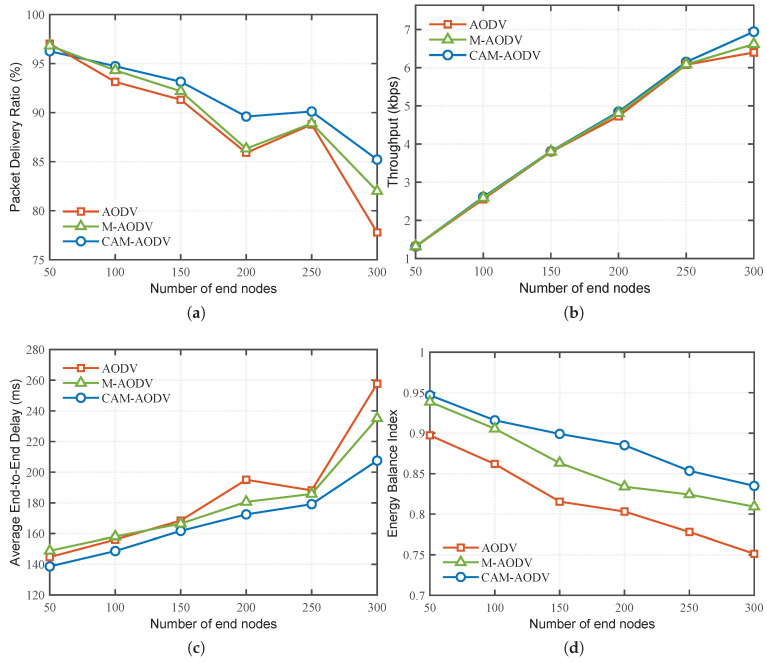
Protocol performance evaluation under different network densities. (**a**) Packet delivery ratio. (**b**) Throughput. (**c**) Average end-to-end delay. (**d**) Energy balance index.

Under a fixed per-node packet generation rate, the number of end nodes is incrementally increased from 50 to 300 in steps of 50 to evaluate the comprehensive impact of network density on protocol performance. The simulation results are illustrated in Figure 15.

In terms of the PDR and system throughput, as illustrated in Figure 15a,b, with the expansion of the node scale, the increased spatial density intensifies co-channel collisions, causing the overall network PDR to exhibit a downward trend. In the 300-node scenario, the traditional AODV protocol utilizes a single hop-count metric, leading to a heavy concentration of traffic at local relay nodes. M-AODV, by incorporating a cross-layer multi-metric mechanism, alleviates this issue to a certain extent, achieving a PDR of 82.00%. In contrast, CAM-AODV effectively achieves traffic load sharing by introducing a congestion-aware metric and a priority-based backoff mechanism. Simulation results demonstrate that in the 300-node scenario, the PDR of CAM-AODV reaches 85.21%, and its throughput exhibits a more stable growth curve.

As illustrated in Figure 15c, the average end-to-end delay experiences a significant surge in traditional AODV when the number of end nodes exceeds 200, caused by MAC-layer queue backlogs at local relay nodes, reaching 257.68 ms in the 300-node scenario. Although M-AODV introduces a cross-layer metric between the physical and network layers, it is constrained by the lack of real-time awareness and coordination mechanisms for the underlying MAC-layer queue status, causing its delay to subsequently increase to 235.10 ms. In contrast, CAM-AODV employs a cross-layer queue length awareness mechanism during the routing decision phase. When the load on core nodes becomes heavy, it proactively diverts data flows to bypass relays with idle channels. This strategy effectively mitigates the network-wide queuing delay. Under the 300-node scenario, the average end-to-end delay of CAM-AODV is reduced to 207.53 ms, representing a reduction of approximately 19.46% compared to the traditional protocol, which is also significantly lower than that of M-AODV.

Regarding energy balancing, the performance comparison across various network densities is illustrated in Figure 15d. As the number of end nodes increases, the network-wide concurrent traffic escalates, causing the Energy Balancing Index (EBI) of all three protocols to exhibit a downward trend. In the 300-node high-density scenario, the traditional AODV protocol relies solely on a single hop-count metric, leading to a heavy concentration of traffic on local backbone links, which reduces its corresponding EBI to 0.7513. Although M-AODV incorporates cross-layer metrics, it employs a fixed energy threshold for packet dropping and lacks continuous dynamic load-balancing capabilities. Consequently, nodes continue to suffer from sustained high energy consumption before reaching the threshold, resulting in an EBI of 0.8093. In contrast, the proposed CAM-AODV protocol eliminates the fixed threshold constraint. During the route discovery phase, it utilizes non-linear energy and congestion penalty mechanisms to steer heterogeneous traffic towards edge relay nodes with more abundant residual energy. This cross-layer dynamic traffic diversion mechanism effectively balances the global energy consumption, enabling the EBI of CAM-AODV to reach 0.8349, which represents an improvement of approximately 11.13% over traditional AODV.

#### 4.2.3. Impact of Network Load on Protocol Performance

**Figure 16:**
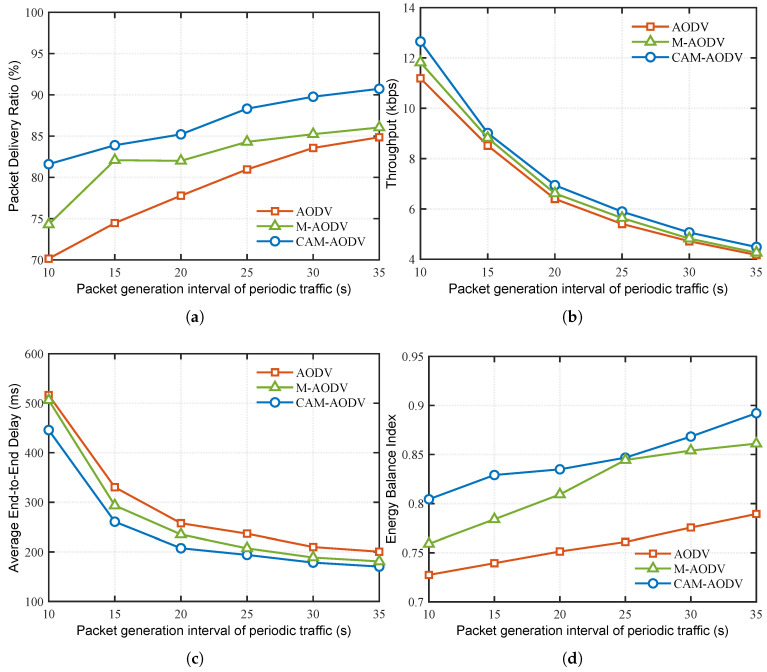
Protocol performance evaluation under different network loads. (**a**) Packet delivery ratio. (**b**) Throughput. (**c**) Average end-to-end delay. (**d**) Energy balance index.

Under a fixed number of end nodes, the packet generation interval of periodic data traffic is varied to evaluate the comprehensive impact of concurrent network load on protocol performance. The simulation results are illustrated in Figure 16.

When evaluating the PDR and system throughput under varying loads, as illustrated in Figure 16a,b, shortening the packet generation interval from 35 s to 10 s causes traditional AODV to suffer from local aggregation of multi-hop traffic, leading to a drop in its PDR to 70.15%. Although M-AODV exhibits improved performance, it still encounters concurrency bottlenecks when faced with bursty heavy traffic, with its PDR decreasing to 74.30%. By employing a multi-path dynamic traffic diversion mechanism, CAM-AODV achieves a PDR of 81.60% under the 10 s high-concurrency scenario, representing an improvement of approximately 16.32% over traditional AODV. Furthermore, while traditional AODV is affected by co-channel collisions that result in an increased physical-layer packet loss rate, CAM-AODV introduces a concurrent collision avoidance mechanism to enhance the transmission efficiency of valid data. Consequently, its system throughput reaches 12.65 kbps, demonstrating superior load adaptability.

Concerning the average end-to-end delay, as illustrated in Figure 16c, a high traffic arrival rate increases the node queue occupancy duration. Traditional AODV is unable to perceive node congestion states, causing packet waiting times at congested nodes to be significantly prolonged, with its delay reaching 516.35 ms under the 10 s interval; although the delay of M-AODV is lower than that of the traditional protocol, it still reaches 506.12 ms. In contrast, CAM-AODV distributes data flows across multiple candidate paths through cross-layer congestion awareness, thereby reducing the queuing delay at core nodes. Simulation results demonstrate that under the 10 s high-concurrency scenario, the average end-to-end delay of CAM-AODV is reduced to 445.82 ms, representing a reduction of approximately 13.66% compared to traditional AODV.

Regarding energy balancing, the performance comparison under varying network loads is illustrated in Figure 16d. Under the 10 s high-concurrency traffic scenario, traditional AODV is unable to perceive network states, causing high-frequency traffic to continuously aggregate along the shortest path. This leads to the rapid energy depletion of core relay nodes, dropping its EBI to 0.7275. Although M-AODV incorporates energy detection, its fixed-threshold packet dropping mechanism struggles to adapt to bursty heavy traffic scenarios and fails to achieve dynamic load balancing, resulting in an EBI of 0.7591. In contrast, the proposed CAM-AODV protocol integrates the MAC-layer congestion state and a non-linear energy penalty mechanism in a cross-layer manner during routing decisions. When core relay nodes experience heavy loads, the algorithm dynamically diverts high-frequency traffic to bypass relays with relatively idle channels and sufficient energy reserves. Consequently, its EBI reaches 0.8044, representing an improvement of approximately 10.57% over traditional AODV.

### 4.3. Model Validation and Limitations

This study employs the 3D Axis-Aligned Bounding Box (AABB) modeling approach to simulate NLOS propagation characteristics in complex environments. However, constrained by practical conditions, large-scale field tests have not yet been conducted to acquire real-world data for calibrating the propagation parameters. Instead, this paper establishes the core loss parameters based on the log-distance path loss theory, integrating the statistical distribution of building penetration loss from the ITU-R P.2109-2 standard [28] and the classical layered cumulative framework of the Multi-Wall Model [27].

Although the current simulation model can effectively evaluate and compare the performance differences among various cross-layer protocol frameworks, routing algorithms, and MAC mechanisms within a controlled network environment, the wireless propagation characteristics at the physical layer are highly susceptible to the coupled effects of multiple factors in real-world scenarios, such as terrain, building blockages, and environmental interference. Consequently, empirical measurement data remains the fundamental basis for validating physical layer propagation models. Building upon the existing research foundation, future work will focus on field tests of LoRa signals in complex scenarios. By utilizing empirical fading data to calibrate the parameters of the 3D propagation model, the calibrated model will then be employed to further optimize the cross-layer architecture and performance evaluation system of LoRa ad hoc networks. Furthermore, subsequent evaluations will be extended to multi-gateway macro-diversity scenarios to investigate the synergistic gains achieved by integrating distributed ad hoc routing with multi-gateway redundant reception mechanisms in ultra-large-scale networks.

## 5. Conclusions

To address the limited coverage and capacity bottlenecks of the standard LoRaWAN star architecture in dense deployment scenarios, this paper broke through the limitations of single-layer protocol optimizations and proposed a distributed LoRa ad hoc network protocol framework encompassing topology deployment and cross-layer synergy. This study verified the feasibility and outstanding advantages of cross-layer joint design. At the physical layer, the hybrid heuristic 3D relay deployment approach eliminated physical communication blind spots caused by NLOS blockages. Relying on the non-preemptive priority access mechanism and the differentiated backoff strategy based on symbol energy detection at the MAC layer, the framework effectively isolated co-channel collisions generated by heterogeneous traffic. At the network layer, the proposed CAM-AODV algorithm integrated underlying link states, MAC layer congestion queues, and nodal residual energy, achieving dynamic traffic diversion and scheduling for highly concurrent traffic across multiple candidate links.

Simulation results demonstrated that the proposed framework exhibited robust performance when facing increased network density and bursty high loads. Compared to traditional AODV and M-AODV protocols, this framework effectively enhanced network transmission efficiency in harsh environments, improving the PDR by 16.32% and reducing the average end-to-end delay by 19.46%. Furthermore, by alleviating localized congestion through a non-linear energy penalty mechanism, the framework improved the EBI by 11.13% and 10.57% under the two evaluation scenarios, respectively, compared to traditional protocols, thereby achieving a balanced distribution of global energy consumption.

Additionally, the designed framework exhibited an asymmetrical distribution characteristic regarding system computational overhead. By effectively offloading complex computing tasks, such as state statistics and parameter calculations, to the central gateway, it consistently maintained the lightweight operational nature of end nodes. This achieved a reasonable balance between network performance optimization and actual deployment and maintenance costs. Constrained by objective experimental conditions, this study did not conduct large-scale physical hardware testing at the current stage. Future research will focus on establishing a real-world wireless testbed to precisely calibrate protocol parameters based on field-collected channel fading data. Concurrently, future work will expand to multi-gateway macro-diversity application scenarios to further explore the synergistic gains and engineering implementation value of this distributed cross-layer protocol in ultra-large-scale IoT systems.

## Figures and Tables

**Table 1:** Comparison of the proposed framework with the existing literature.

Reference	Topology and Environment Modeling	MAC Layer Anti-Collision Optimization	Routing Metric Mechanism	Support for Heterogeneous Traffic
Ref. [5]	2D plane	Not addressed	Single link SNR	No
Ref. [7]	Not addressed	LBT mechanism	Not addressed	No
Ref. [10]	Not addressed	Semi-distributed scheduling for bursty traffic	Not addressed	Yes, bursty traffic only
Ref. [12]	Not addressed	Not addressed	Probabilistic forwarding and minimum hop count	No
Ref. [14]	Not addressed	Not addressed	RSSI and congestion level	No
Ref. [23]	2D plane	Not addressed	Multi-dimensional states such as trust value and residual energy	Yes, hybrid IoT services
Proposed framework	3D complex building penetration model	Time-domain isolation based on symbol energy detection and differentiated contention windows	Deep coupling of link quality, congestion level, and residual energy	Yes, strict isolation of multiple priorities

**Table 2:** Network simulation parameter settings.

Parameter Name	Parameter Value
Scenario space size/m^3^	923×498×25
Number of end nodes	50–300
Number of gateway nodes	1
Number of relay nodes	Dynamically deployed based on coverage algorithm
Channel bandwidth (BW)/kHz	125/500
SF	7–12
Coding rate (CR)	4/5

**Table 3:** Heterogeneous traffic types and priority scheduling parameter settings.

Traffic Type	Data Flow Direction	Priority	Payload
Emergency alarm traffic	End node → Gateway	1	32 Byte
Control signaling traffic	Gateway → End node	2	24 Byte
Periodic data traffic	End node → Gateway	3	64 Byte

## Data Availability

Data are contained within the article.
